# OSCAR: An Optimized Scheduling Cell Allocation Algorithm for Convergecast in IEEE 802.15.4e TSCH Networks

**DOI:** 10.3390/s21072493

**Published:** 2021-04-03

**Authors:** Mohamed Osman, Frederic Nabki

**Affiliations:** Department of Electrical Engineering, École de Technologie Supérieure, Université du Québec, Montréal, QC H3C 1K3, Canada; frederic.nabki@etsmtl.ca

**Keywords:** wireless sensor network, TSCH, convergecast scheduling, RPL, MAC, low latency, low power

## Abstract

Today’s wireless sensor networks expect to receive increasingly more data from different sources. The Time Slotted Channel Hopping (TSCH) protocol defined in the IEEE 802.15.4-2015 version of the IEEE 802.15.4 standard plays a crucial role in reducing latency and minimizing energy consumption. In the case of convergecast traffic, nodes close to the root have consistently heavy traffic and suffer from severe network congestion problems. In this paper, we propose OSCAR, an novel autonomous scheduling TSCH cell allocation algorithm based on Orchestra. This new design differs from Orchestra by allocating slots according to the location of the node relative to the root. The goal of this algorithm is to allocate slots to nodes according to their needs. This algorithm manages the number of timeslots allocated to each node using the value of the rank described by the RPL routing protocol. The goal is that the closer the node is to the root, the more slots it gets in order to maximize the transmission opportunities. To avoid overconsumption, OSCAR sets up a mechanism to adjust the radio duty cycle of each node by reducing the slots allocated to inactive nodes regardless of their position in the network. We implement OSCAR on Contiki-ng and evaluate its performance by both simulations and experimentation. The performance assessment of OSCAR shows that it outperforms Orchestra on the average latency and reliability, without significantly increasing the average duty cycle, especially when the traffic load is high.

## 1. Introduction

A WSN is a network made up of small, resource-limited devices that communicate wirelessly in a network. Each device, called a sensor node, operates with other devices in the network to perform certain operations, such as monitoring the environment (temperature, sound, pressure, etc.). Over the past ten years, there has been an explosive growth in the number of Internet of Things (IoT) devices [1]. From the car to the mobile phone, to watches and televisions, connected devices are omnipresent in everyday life, and continue to expand their footprint [2]. Wireless sensor networks (WSNs) are an important part of this trend with numerous applications (home automation, industrial automation, video games, health monitoring, etc.) [3]. The Time Slotted Channel Hopping (TSCH) [4] protocol defined in the IEEE 802.15.4-2015 version of the IEEE 802.15.4 standard orchestrates the medium access for WSNs according to a time-frequency communication schedule. MAC protocols play a crucial role to meet the energy and latency requirements of these networks. As the number of connected objects increases, the volume of data generated by the Internet of Things greatly increases. A large amount of data passes through these nodes and raises challenges. Among these, the critical issue in WSN is energy consumption and latency especially for industrial applications [5,6].

An important problem with wireless communications is the minimization of the energy consumption while ensuring latency and that every bit of information transmitted over the network is not lost. Energy consumption is one of the biggest concerns as a sensor must be able to operate without intervention or battery replacement for months or even years. Indeed, users generally choose to deploy these sensor nodes randomly in a targeted area, often difficult to access, in large numbers (hundreds or even thousands of sensor nodes), so their replacement is practically impossible or a very expensive task. Latency in TSCH networks is also crucial in many applications such as industrial automation, and reliability is important so that all data are received at the final destination. The TSCH MAC layer plays a crucial role in meeting power consumption and latency requirements. More specifically, the cell scheduling algorithms that are responsible for node behavior at each timeslot are at the center of these parameters.

A certain number of studies, which aims at the development of these algorithms, have emerged to try to answer these different challenges. These scheduling algorithms can be classified either as centralized [7,8,9], distributed [10,11,12,13], or autonomous [14,15,16,17]. Centralized scheduling allows a central entity collect all the information of the network, then it calculates the link schedules. However, when the network topology changes, the update of the network information is done more slowly by the root, which becomes overloaded, and therefore can be inefficient.The distributed approach is characterized by the fact that the schedule is built through negotiations between neighboring nodes. However, this negotiation may cause a scheduling delay due to additional overhead. Autonomous scheduling is an attractive alternative as it avoids these problems by allowing each node to autonomously choose its own schedule without any signaling for schedule creation or modification.

Orchestra [14] is the pioneer of this autonomous approach. However, despite several strengths such as the use of simple scheduling rules and the high delivery ratio, Orchestra determines the TSCH schedule for each node regardless of its traffic load, which can significantly affect communication delay. The demand of each node is not the same and varies depending on the location of the node in the network. For example, nodes close to the root are constantly in demand and have heavy convergecast traffic because all of the nodes transmit packets to this destination.

In this paper, we propose an optimized scheduling cell allocation algorithm (OSCAR), which is an autonomous scheduling TSCH cell allocation algorithm based on Orchestra to address the aforementioned issues. This new design differs from Orchestra by allocating slots according to the location of the node relative to the root. The goal of this algorithm is to allocate slots to nodes according to their needs. Indeed, Orchestra determines the TSCH schedule for each node regardless of its traffic load, which can significantly affect communication latency and energy consumption. There is therefore an accumulation of packets in the queues of nodes close to the root due to their high load, which can lead to a loss of packets induced by an overflow of the buffer memory. OSCAR is characterized by the classification of nodes according to the distance from the root. Each node is assigned a class ID, representing the layer it occupies based on the geographic location relative to the root. Moreover, OSCAR provides a method to adjust the radio duty cycle of each node and improve energy efficiency by turning off the radio when no packets are received. In this work, OSCAR is validated through simulations and experimentation. The performance analysis is first carried out on a test bench composed of 11 CC2650 Launchpad nodes and then a simulation is used to evaluate its performance in a larger-scale network. The contributions of this work are summarized as follows.

- A classification architecture for the nodes allowing better distribution of allocated cells according to their position.
- A system to reduce the cells allocated to inactive nodes.

These contributions allow an improvement of the energy and latency metrics of the nodes in the considered scenarios.

The remainder of this paper is organized as follows. Section 2 provides an overview of related works. Section 3 presents the high traffic load issues in convergecast traffic and Orchestra limitations. Section 4 describes the detailed design and implementation of OSCAR. In Section 5, we evaluate the performance of OSCAR and compare it to Orchestra using a test bench and the scenarios considered. Section 6 provides a discussion of the results and future research directions. Finally, the conclusion is presented.

## 2. Related Work

An aspect not specified in the IEEE 802.15.4 standard is the construction of a TSCH schedule. Therefore, this task has attracted great attention from the research community.

In centralized schemes, a central entity collects the entire network information and then calculates the link schedules. TASA [7] is a centralized approach where the schedule is built by a single node. This scheduling builds time/frequency patterns based on the network topology graph and the traffic load at each node, while reducing latency and radio duty-cycle at the same time. The CLS [8] algorithm constructs efficient multi-hop schedules using a minimal number of centralized control messages, because it allocates and deallocates slots without rescheduling the entire schedule every time. CLS focuses on the reduction of idle listening, while addressing bandwidth requirements of the network. In [9], the authors proposed another centralized raw data convergecast scheduling, called Multichannel Optimized Delay time Slot Assignment (MODESA), which takes into account the availability of multiple channels to reduce the TDMA cycle length while ensuring a fair medium access. It sorts the nodes according to their priorities, where the highest priority corresponds to the highest number of remaining packets in one node’s buffer. Although these approaches present a theoretical scheduling which seems efficient, actually, the disadvantage of centralized scheduling lies in the change of the topology. In this case, the update of the network information is done more slowly by the root, which becomes overloaded. This is why this type of centralized approach is recommended when the topology does not change much and therefore the environment is static.

Then comes the distributed approach, which attempts to allocate cells through a procedure for establishing connections between neighbors. The distributed scheduling is more flexible when faced with sudden network changes than the centralized schemes, which collect information from the central entity and determine the schedule. DeTAS [10], one of them, is a distributed version of TASA that targets networks with several sink nodes. This distributed scheduling is a traffic-aware algorithm that builds the schedule based on the traffic generated by each source node where the network is modeled as multiple routing graphs, each rooted at a different sink. Wave, another distributed scheduling algorithm that schedules nodes in successive waves, is proposed in [11]. In each wave, each node having a packet to transmit is assigned a time slot and a channel. The sink node sends a message to its children to trigger the computation of the first wave. The particularity of this algorithm is that it minimizes the slotframe size required for the convergecast. Each slotframe is divided with a unit called the wave and generates waves repeatedly to form a schedule for all nodes in the network to transmit packets generated at regular intervals to the sink. In [12], DeAMON, which is decentralized adaptive multi-hop scheduling protocol for 6TiSCH wireless networks, is introduced. This distributed scheduling for industrial monitoring and control applications offers solutions for upward traffic only and does not consider downward traffic. DIVA, a distributed divergecast scheduling algorithm, is presented in [13]. It is a completely distributed scheduling algorithm for divergecast traffic where nodes depart traffic to all neighbor nodes, as opposed to convergecast where leave nodes concentrate traffic towards the root node. Connections to neighbor nodes are established according to random opportunities independent of the network traffic rate at each node. Fundamentally, the distributed approach has weaknesses such as the need to negotiate between neighbors, which can lead to scheduling delay due to additional messaging.

In autonomous scheduling, each node in the network determines the communication scheduling as the distributed approach. However, unlike distributed scheduling, each node can autonomously choose its own schedule without any signaling for schedule creation or modification. Autonomous scheduling has the advantage of less overhead in the network for scheduling than centralized and distributed approaches. The publication of Orchestra [14] initiated the field of autonomous scheduling for TSCH. Orchestra is a recent scheduling solution for TSCH which brings important advantages such as the use of simple scheduling rules with a high delivery rate. Orchestra has several strengths that make it a promising scheduling solution for TSCH networks. Orchestra scheduling is neither centralized nor distributed and is calculated autonomously, where each network node locally maintains its own schedule based on information from the routing layer. The Orchestra schedule consists of three types of slotframes: The EB slotframe, which has a size of 397 timeslots, is dedicated to EB (TSCH Enhanced Beacon) transmissions, and is used for TSCH association and child–parent synchronization. The broadcast slotframe, which has a size of 31 timeslots, is intended for broadcasting control messages and for transmitting any packet when a unicast cell is not planned. Finally, the unicast slotframe is intended for the transmission of traffic between the RPL parent and all of its children. Escalator [15], another autonomous scheduling scheme which is based on Orchestra, generates a consecutive timeslot schedule along the packet forwarding path to minimize the packet transmission delay. This means that in one timeslot, a node receives a packet generated from a descendant node, and in the next timeslot, the node sends the received packet to its parent immediately. On the other hand, ALICE [16] allocates a unique cell for each traffic direction link instead of allocating a cell for each node. This strategy has helped reduce contention, collision, and latency issues. e-TSCH-Orch [17] also tried to reduce Orchestra latency by focusing on scheduling. It operates such that when a node sends a packet to a neighboring node, it indicates the number of packets in its transmit queue. Its neighboring node schedules this amount of consecutive Rx cells, and when the slotframe has enough free time slots, the node can use them to empty its queue. TESLA [18] is an adaptive approach that aims to minimize energy consumption without sacrificing reliable packet delivery by utilizing incoming traffic load to estimate channel contention level experienced by each neighbor. It periodically self-estimates the contention level of the neighbors based on the traffic load and adjusts its slotframe size. The limitation of setting slotframe size as a fixed global constant is addressed by periodically adjusting the slotframe. In TESLA, each node continuously sends its traffic load to its neighbor nodes exchanging control packets so that all nodes in the network can figure out their neighbors’ traffic loads, and add or remove slots from the slotframe continuously. Parametrized Adaptive and Autonomous Scheduling (PAAS) [19] is another autonomous and distributed algorithm, and it works adaptively to traffic intensity, slotframe length, and reliability requirements. Nodes using PAAS exchange useful information for scheduling and allocates more cells if they are needed. Deciding which schedule is a good one actually depends on how high the traffic intensity is. In [20], the authors show that Orchestra has problems when operating under limited link capacity conditions, especially in regions close to the network’s root node. To this end, the authors of [20] design and evaluate a special Orchestra scheduler rule for the root node. This rule adds a dedicated slotframe for communication between the root node and the nodes directly adjacent to the root node. The option to have multiple root nodes in a single TSCH network is also introduced. A network node is expected to install the root slotframe if it has any root nodes as direct neighbors. Experimental results in a testbed and real-world deployment show that the network provides reliable and energy-efficient improvement.

The autonomous approach is currently a significant focus of researchers, in particular because of its advantages over the other two aforementioned approaches. Accordingly, it is the approach studied in this work.

## 3. High Traffic Load Issues

### 3.1. Congestion and Queue Overflow in Convergecast Traffic

Networks with convergecast traffic, such as those used for data collection applications, suffer from severe congestion problems [21]. These applications often have a convergecast behavior, where all of the data collected by all of the sensors in the network are routed to the sink. With high traffic load, the number of data packets traveling from leaf nodes towards the sink becomes higher, which leads to congestion mainly in nodes located near the sink. Congestion has negative consequences on network performance such as delay, queue overflow, and, ultimately, packet loss. When a network is congested, it is not simple for nodes to access the medium and transmit their data toward the sink because of high contention and collisions. In order to mitigate the impact of such congestion, a better distribution of the cells allocated to the nodes is necessary according to the node position within the network. In 802.15.4e networks with TSCH mode, a node *n* can transmit two types of data: periodic packets denoted by $A_{l}^{W}\left(n\right)$ and event packets denoted by $A_{l}^{V}\left(n\right)$.

The total flow generated in a timeslot *T* by a node *n* denoted by $A_{l}^{T}\left(n\right)$ is given by [22]

(1) $$A_{l}^{T}\left(n\right)=A_{l}^{W}\left(n\right)+A_{l}^{V}\left(n\right).$$

The distribution of the number of data packets N(t) generated by each node in the network from the start of the slotframe to its end is calculated as follows [22]:

(2) $$P\left\{N\left(t\right)=n\right\}=\frac{{\left(\lambda t\right)}^{n}}{n!}e^{\lambda t}.$$

We will assume that the queue associated with link *l* is denoted by $Q_{l}\left(t\right)$ and $E_{l}\left(t\right)$, the amount of data transmitted over a link *l* in timeslot t. With $E_{l}\left(t\right)\leq Q_{l}\left(t\right)$ as a constraint, the evolution of the traffic queue for a specific node is then given by [23]

(3) $$Q_{l}\left(t+1\right)=Q_{l}\left(t\right)+A_{l}\left(t\right)-E_{l}\left(t\right).$$

In [23], a constraint where the amount of data transmitted over a link must not exceed the queue to that link was taken in order to not have negative values in the queue evolution equation. $A_{l}\left(t\right)$ denotes the amount of data generated by node n in timeslot t. The root maintains no queue as it is the destination node for all flows. The idea would therefore be to ensure that the nodes close to the root exhibit as little congestion as possible.

### 3.2. Orchestra Limitations

The current TSCH scheduling does not support dynamic allocation of cells based on traffic and node requirements. Despite its autonomous characteristics, Orchestra [14] determines the TSCH schedule for each node regardless of its traffic load, which can significantly affect communication delay. In the Orchestra schedule, each node reserves slots per slotframe, independently from the amount of data traffic it has to deliver to the root. This is a problem because the demand of each node is not the same and varies depending on the location of the node in the network. Nodes close to the root are constantly in demand and have heavy traffic because all nodes transmit packets to this destination. These nodes close to the root, in addition to sending packets themselves, also forward packets from the leaf nodes to the root node. This results in an accumulation of packets in the queues of nodes close to the root due to their high load, which can lead to a loss of packets induced by an overflow of the buffer memory [23]. On the other hand, the nodes located at the end of the network do not transit that much traffic, and their scheduling can be relaxed. This is why a better allocation of cells is necessary. This limitation makes Orchestra impractical for many delay-sensitive applications. The limitation is that when traffic is not distributed evenly across the network, Orchestra does not adapt its cell allocation mechanism. This limitation is due to the fact that Orchestra allocates the same number of timeslot to all nodes, which leads to significant delays, particularly near the root node where the traffic is dense due to the queuing of packets in the buffers of congested nodes [17]. Consequently, each of these nodes close to the root requires, compared to the leaf nodes, a greater number of available slots to be able to clear this congestion of traffic. We must therefore try to minimize the latency at this level and give these nodes more resources to dump traffic in order to improve the convergecast performance. Indeed, several critical applications such as monitoring and alerting applications may suffer from these delays caused by high traffic load.

## 4. Oscar: Tsch Optimized Cell Allocation Algorithm

In this section, we present the detailed design and implementation of the proposed TSCH optimized scheduling cell allocation algorithm.

### 4.1. Design

#### 4.1.1. Rpl Rank

The concept of rank introduced by the Routing Protocol for Low Power and Lossy Networks (RPL) [24] is used as the basis of OSCAR to determine the distance from the root. Usage of the rank within the OSCAR algorithms will be described in later subsections.

RPL is a proactive remote vector routing protocol for IPv6-based LLNs. The RPL topology is organized in a Destination Oriented Direct Acyclic Graph (DODAG) structure. Besides the peripheral memory, the number of nodes in a DODAG is not limited. Typically, the DODAG root corresponds to the IPv6 edge router which connects the nodes to the outside world (i.e., Internet or any other network) and from which it can receive commands for the management of the collected data. Each node is associated with a rank that is calculated using the Minimum Rank with Hysteresis Objective Function (MRHOF). The concept is that the closer the node is to the root, the smaller the rank. A node sends a packet to the root by forwarding it to a neighbor node of lower rank. Each node within the network has an assigned rank, which increases as the node move away from the root.

The establishment and maintenance of the DODAG are ensured by DODAG Information Object (DIO) control messages broadcast using a hold timer. DIO packets contain information such as metrics used to calculate the cost of the path. Initially, only the DODAG root is part of the active RPL topology. Subsequently, DIO messages periodically broadcast the configuration parameters like the DODAG-ID and its rank within its neighborhood. As soon as a node receives multiple DIOs from different sources, it selects one of them as its preferred parent (according to rank) which also acts as the next hop to reach the DODAG root. RPL also defines the Destination Advertisement Object (DAO), which is an ascending message allowing a node to announce its prefix for the establishment of downward routes. Therefore, for example, when changing the preferred parent due to link variation, a node sends a DAO upstream to the new preferred parent to configure a new downlink route. To ensure that the message has been received, a DAO-Ack is sent by the parent to the child node.

#### 4.1.2. Rescheduling Algorithm

The purpose of Algorithm 1 is to grant slots to the nodes according to their requirement. We know that the nodes close to the root are those with a high traffic load. In this vision, this algorithm is part of the scheduling of these nodes and proposes to manage the number of timeslots allocated to each node using the value of the node’s rank. On initialization, the rank value is extracted for each node of the network. Classes are determined based on the rank value in RPL.

RPL networks introduce the concept of rank to define the individual position of a node relative to all other neighbors and relative to the root. OSCAR is characterized by the classification of nodes in 6 classes according to their rank value knowing that the rank of the root is equal to 0. Each class is delimited by rank values. Each node is assigned a class ID, representing the layer it occupies. The class ID, which ranges from 0 to 5, is set according to the distance from the root. In the OSCAR configuration presented here, any node whose rank is between 0 and 128 is in class 0. For class 1, the node rank must be between 128 and 256, class 2 between 256 and 384, class 3 between 384 and 512, and class 4 between 512 and 768. Finally, any node with a rank greater than 768 is in class 5. Therefore, nodes located in class 4 are more distant from the root compared to those located in class 2. The well-known trade-off between energy and latency has been a challenge to be taken into account when designing of OSCAR. We chose to use 6 classes because this number presented a good trade-off in terms of energy/latency balance. Depending on network topologies and traffic types, different number of classes and rank threshold could be configured. The timeslot allocation mechanism of OSCAR generates a timeslot schedule for the slotframe that uses the routing topology information, the Class ID and the node ID. To explain the slot allocation process, we first discuss the partitioning strategy. This is a mechanism for the distribution of timeslots over the next six slotframes. If the node belongs to class 0, it will have 6 slots on the next six slotframes. Class 1 will have five slots on the next six slotframes and so on up to the last class which will have only one slot on the six slotframes.
**Algorithm 1:** Reschedule cell based on class*Classes definition and initialization of the current class*;**Input**: New Class**if***New_Class = Current_Class***then**((| return;**end**(**if***New_Class < Current_Class***then**(| Allocate_more_slots();**else**((| Reduce_allocated_slots();**end**(

**Figure 1:**
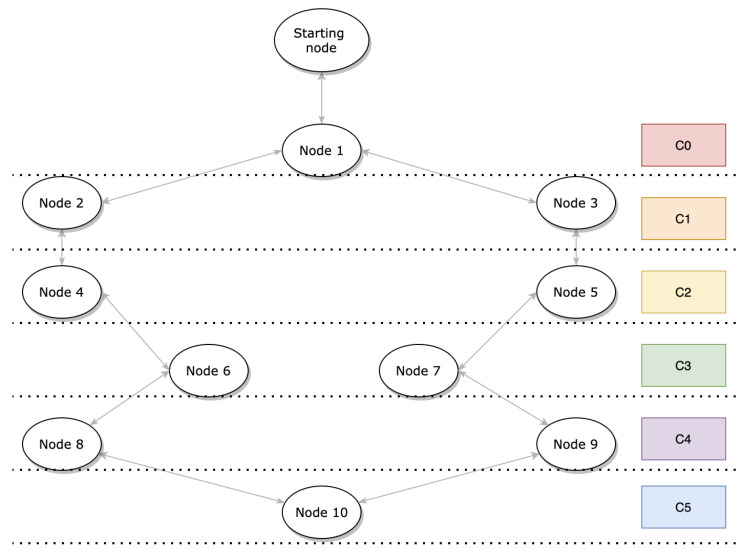
An example of classification of the network topology and the first network topology used in the performance evaluation.

When the rank changes, OSCAR allocates or removes a slot according to the new value of that rank. Therefore, by moving the node away from the root, fewer slots will be allocated. Conversely, more slots are allocated when the node is moved closer to the root. Thus, any node which changes position in the topology of the network sees its class number changed and consequently the number of slots allocated to it. The rescheduling algorithm occurs every five packets, i.e., there is a refresh of the slot allocation and the current state of the network every five packets. The Allocate_more_slots function allocates a slot according to the rank value. For example, if the node is originally located in class 5 and then ends up in class 3, the function allocates two additional time slots. The Reduce_allocated_slots function, on the other hand, removes a slot according to the value of the rank. With the same example, if the node is originally located in class 3 and then ends up in class 5, the function removes two of the added time slots. Nodes listen for any traffic in one slot, and the children maintain a transmit slot towards their parent. Therefore, with this algorithm, each Tx slot is synchronized with an Rx slot at the parent side, so that when a parent Rx slot is removed to diminish its energy consumption, the corresponding Tx children slots are also deleted. It is the parent who therefore sends a packet to all of its children to indicate that the slot is no longer available. On the other hand, when an Rx slot is added to meet a need due to increased traffic, the corresponding Tx slots are re-established. Similarly, the parent sends a packet to all of its children to inform them that the slot is available again. Figure 1 shows an example of the classification of nodes according to their distance from the root. It shows the network consisting of 11 nodes distributed into 6 classes used as an example in this work. The network is composed of a root and 10 nodes distributed in a tree topology.

**Figure 2:**
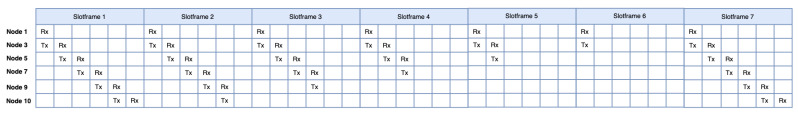
An example of timeslot allocation of the proposed solution.

During each time interval, the nodes all wake up to transmit or receive packets according to their class ID. In addition, different channels are used by two pairs of adjacent layers in order to mitigate interference. The allocation of the number of slots is done as described in Figure 2. For example, in normal Orchestra operation which has a slotframe size of six, node X will have slot numbers 1, 7, 13, 19, 25, 31, and 37. With the proposed implementation in OSCAR, node X keeps its slots as 1, 7, 13, 19, 25, 31, and 37 only if it is located in class 0. However, if it is located in class 1, it will only have slots 1, 7, 13, 19, 25,…, 37. Slot 31 is removed because it is a class 1 node and does not belong to the node closest to root. If node X was class 2, two of the six slots would be removed, i.e., slots 25 and 31, and so on as the class number increases.

For the nodes that are far from the root where the rank is important, less energy will be consumed because less slots will be allocated. For those closest to the root, there will be more consumption, but it is minimal. The other advantage of this mechanism is that it does not cause network congestion as the nodes closest to the root, improving latency.

#### 4.1.3. Duty Cycle Adjustment Algorithm

Each node in the system operates on a duty cycle. Each interval is made up of sleep and wake times. In sleep time slots, the node turns off its radio, and in wake up time slots, the node turns on its radio to transmit or receive packets. With Orchestra, regular reception and transmission time slots are clearly defined and included in the unicast slotframe. All nodes in the network exhibit a 100% duty cycle within the time slots assigned to them to receive. This is not particularly efficient in terms of energy. The idea here is that in the absence of data to receive, a node does not switch on its radio.

Algorithm 2 provides a method to adjust the radio duty cycle of each node and improve energy efficiency. This establishes a complementary mechanism to reduce the allocated slots so that the node turns off its radio even when a node has a good rank. This is done by downgrading the node progressively (class by class). For example, if node X which is in class 1 does not present any activity for a period of time, it is switched to class 2 then to class 3 if the packet exchange does not resume and so on until class 5. Consequently, it benefits from fewer slots allocated progressively. Subsequently, once activity resumes on this node, i.e., a packet is detected to have been received or sent, the initial slot number calculated on the basis of the rank is restored using the rescheduling algorithm.
**Algorithm 2:** Duty cycle adjustment
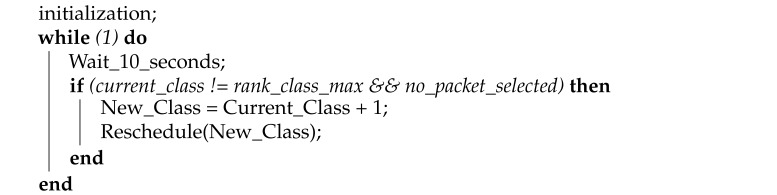


**Figure 3:**
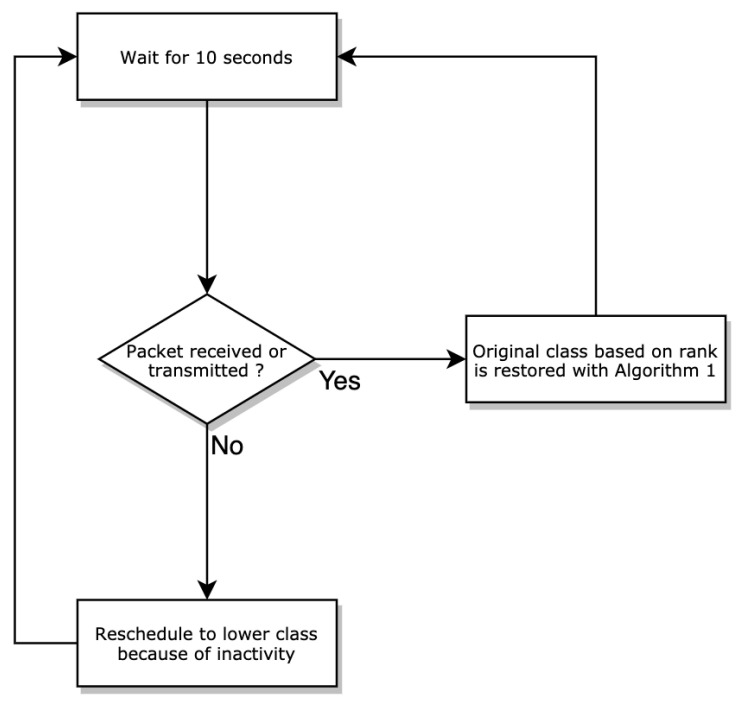
Algorithm 2 design flow graph.

This process is done using a timer that is triggered every 10 s and which monitors the activity of each node, namely, the packets received and sent. Basically, when a node’s timer expires and there are no activities, fewer slots are gradually allocated. The main advantage of this mechanism is that it considerably reduces the energy consumption of the network. The goal is to minimize the energy consumption of the entire network with the constraint that the end-to-end delay expected from each node is as short as possible. The length of a slotframe in OSCAR introduces a trade-off in the end-to-end delay and energy consumption. The shorter the slotframe, the more often nodes have to wake up to listen or transmit, resulting in a higher energy baseline. Notably, shorter slotframes have their slots repeat more often, resulting in a higher traffic capacity and a lower end-to-end delay when approaching the sink. With this algorithm, when traffic is heavy, the size of the slotframe remains the same to support demand and reduce delay. However, once the traffic decreases, the size of the slotframe begins to increase, resulting in a reduction of the energy usage. The flow graph presented in Figure 3 illustrates the process of Algorithm 2 to address network energy requirements.

## 5. Performance Evaluation

In this section, we present the performance of OSCAR. We compare the performance of OSCAR with Orchestra using metrics that we will describe in this section. To evaluate our proposed solution, we have developed and considered two different scenarios:

- The first scenario is to modify the position of the nodes to see the impact that the location has on performance.
- The second scenario aims to measure the impact of the traffic load on the performance of OSCAR.

The following results were carried out on a test bench. The performance of the different metrics in a network consisting of 10 nodes and a root are shown below.

### 5.1. Setup

In this work, the Contiki-NG operating system, an open source platform for IoT which is a fork of contiki [25], was chosen. We measure the performance of OSCAR by comparing it to Orchestra. In practice, the RPL rank information is retrieved via the contiki system. The algorithm in this work does not modify the RPL protocol. The contiki system integrates the RPL protocol and provides facilities to retrieve the necessary information from RPL every five packets. The size of the EB slotframe and the broadcast slotframe remains unchanged with 397 and 31 slots, respectively. The unicast slotframe, on the other hand, has a size of 6 slots for OSCAR and 16 slots for Orchestra. For OSCAR, this means that if we take a portion made up of 36 slots with a size 6 for the unicast slotframe, class 0 will benefit from 6 slots out of the 36 slots, i.e., the next 6 slotframes, so 1 slot every 6 slots. Classes 1–4 will, respectively, obtain on average 1 slot every 7.2, 9, 12, and 18 slots. Finally, the last class 5 will have 1 slot out of the 36 slots. In our experiments, we have deployed a network composed of 11 CC2650 Launchpad boards representing a root and 10 nodes distributed in a tree topology. The root is located with a rank equal to 0. The transmit power is set to 5 dBm where one node acts as the border router. We generate an upward traffic of 1 pkts/min in a convergeast way where each node sends 2000 packets to the root, which means that all nodes forward packets to the sink. Each scenario was performed at least twice for both experiments and simulations to ensure that the results were repeatable. A small variation between repetitions of 2 to 5% was observed for all of the scenarios presented in this work. Depending on the number of nodes in the network, we considered that a unicast slotframe is made up of 6 timeslots. The duration of each timeslot is fixed at 10 ms as indicated in the TSCH 802.15.4e standard. All the tests we have done compare performance between our architecture and orchestra in terms of reliability, low energy consumption, and latency. The goal is to briefly test the behavior of our architecture and its implementation in a wireless sensors network.

### 5.2. Experimental Study

#### 5.2.1. Impact of Node Positions

In this part, two network topologies have been set up to test the impact of the position of the nodes on the performance of the network.

**Figure 4:**
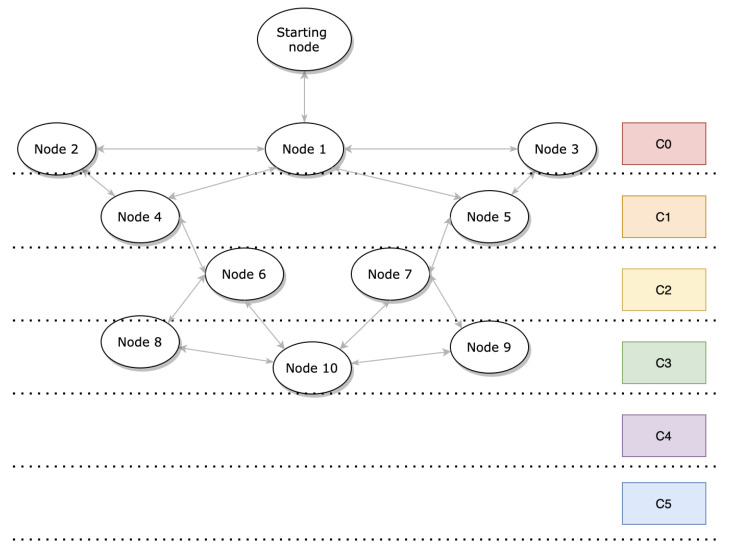
Second network topology used in the performance evaluation.

OSCAR is flexible because the rank delimitations for each class are readily configurable. Accordingly, the impact of the physical position of the nodes was not a focus of this work. Instead, the impact of different link configurations and initial node class groupings on the performance of OSCAR was investigated. The first and second topologies are those illustrated, respectively, in Figure 1 and Figure 4. In both cases, the nodes are distributed over different layers depending on their proximity to the root. Thus, the second form of network is more condensed and close to the root, while the other is more spaced-out and distant from the root.

**Figure 5:**
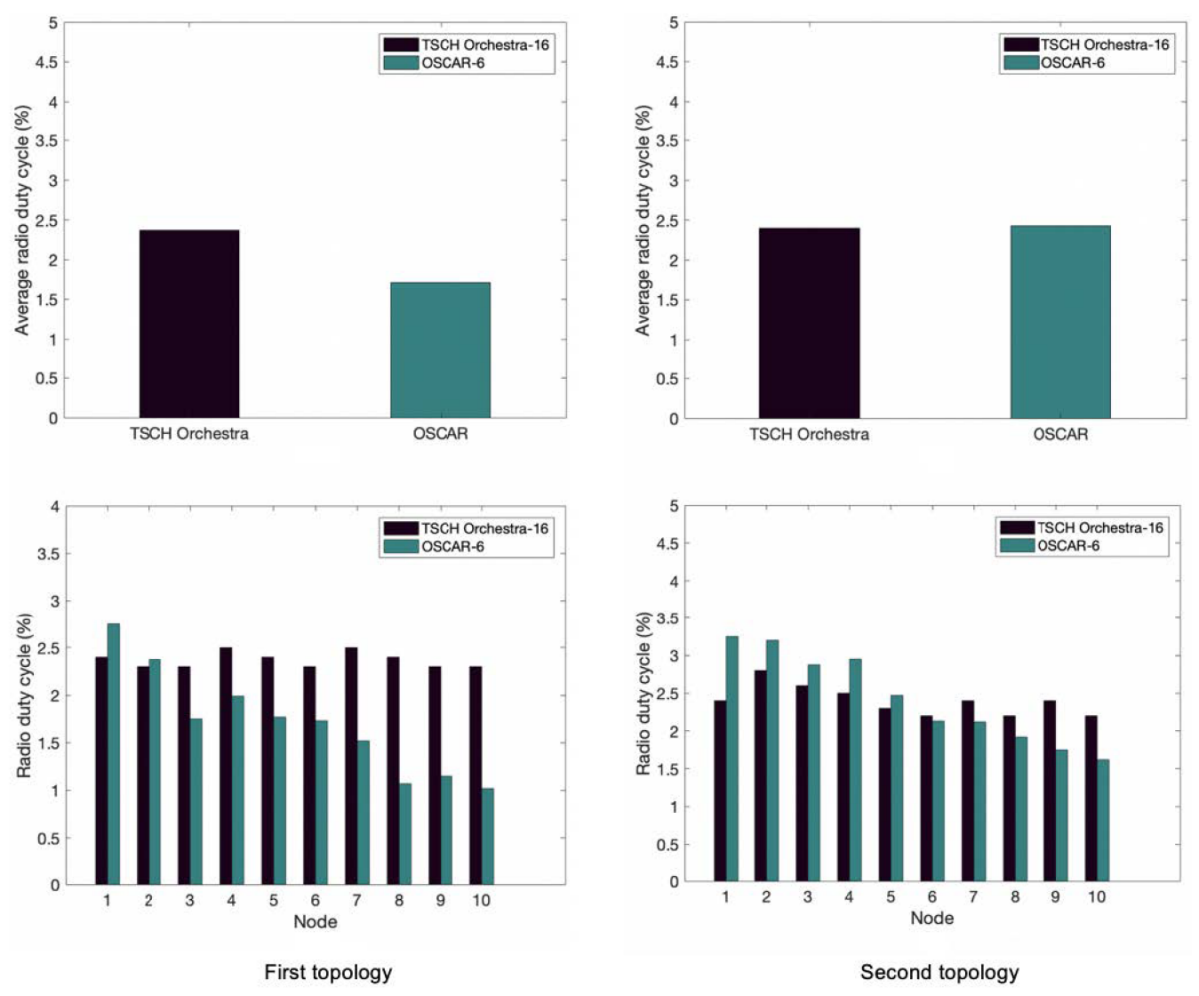
Average duty cycle of OSCAR and Orchestra for the first and second topology.

The first metric tested is the radio duty cycle. Figure 5 shows the time period during which the node is active for the first topology. In this figure, we can see that OSCAR’s average service cycle is lower than that of Orchestra. For OSCAR, nodes 1–3 correspond to those most in demand because they benefit from a larger number of allocated slots and are therefore more active. While nodes 8–10 are the least in demand and thus least active. As such, the cell scheduling mechanisms efficiently use slot allocation to remove slots from inactive nodes. Orchestra in contrast has a relatively flat radio duty cycle for all nodes, as it does not modulate slots allocation.

The result for the second topology is also shown in Figure 5, indicating a slightly higher average duty cycle compared to the first arrangement. It is roughly equivalent to Orchestra’s average duty cycle and is due to Algorithm 2’s duty cycle adjustment. This is explained by the fact that the nodes benefit from a greater number of allocated slots due to their position close to the root node.

**Figure 6:**
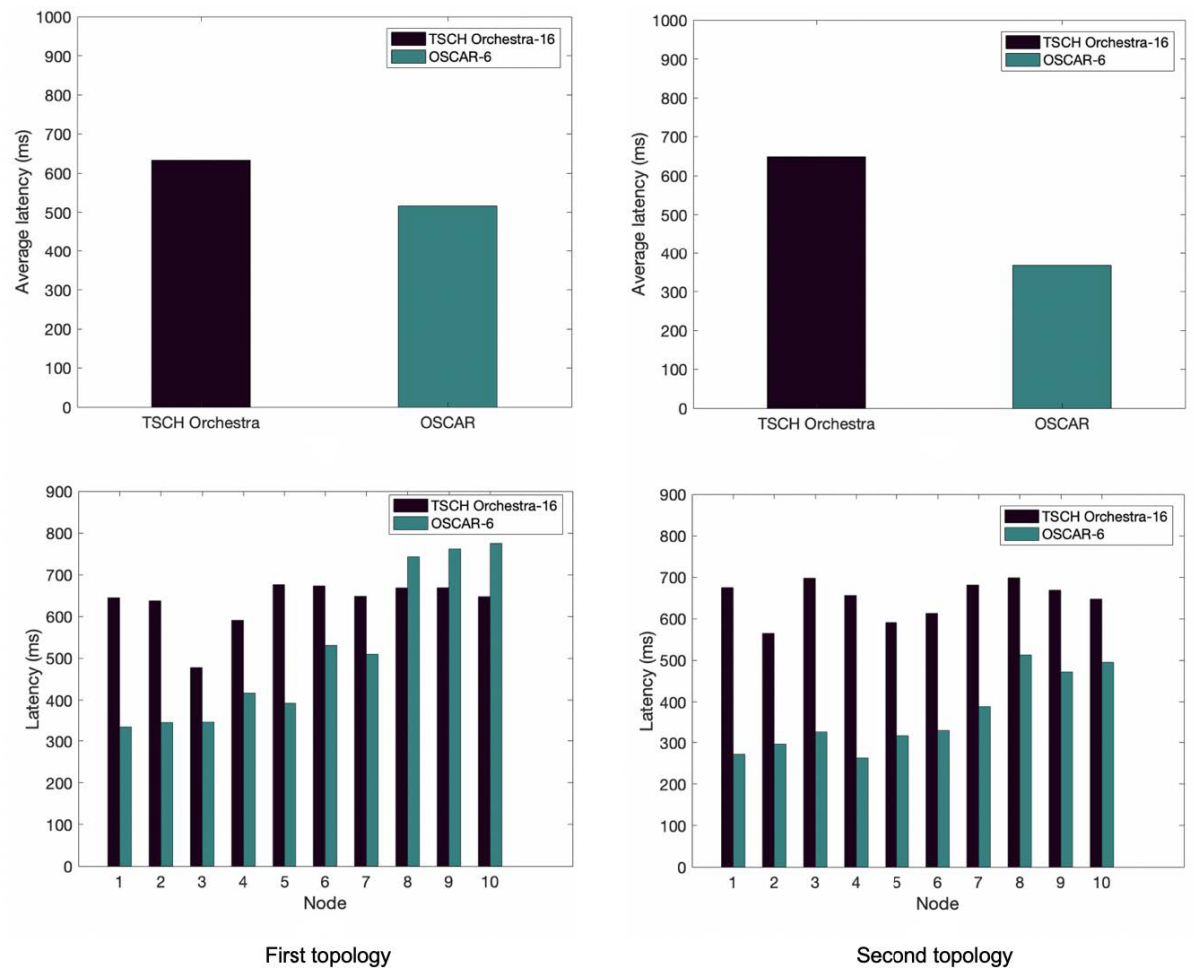
Average end-to-end delay of OSCAR and Orchestra for the first and second topology.

Figure 6 illustrates the results obtained in terms of latency for the first and second topology, respectively. We can see that OSCAR’s average latency is smaller than Orchestra’s in Figure 6. We notice that most nodes show less latency except classes 4 and 5 (i.e., nodes 8–10), a slightly greater end-to-end delay is present. Nonetheless, the global impact of this delay profile results in a reduced average latency, and the nodes close to the root have half the latency. Regarding the second arrangement, OSCAR again exhibits less average latency than Orchestra and the performance gain becomes greater for the first nodes in class 0. The average latency is 42% shorter. This is explained because these nodes have more opportunities to dump traffic, which can solve the conflict problem.

**Figure 7:**
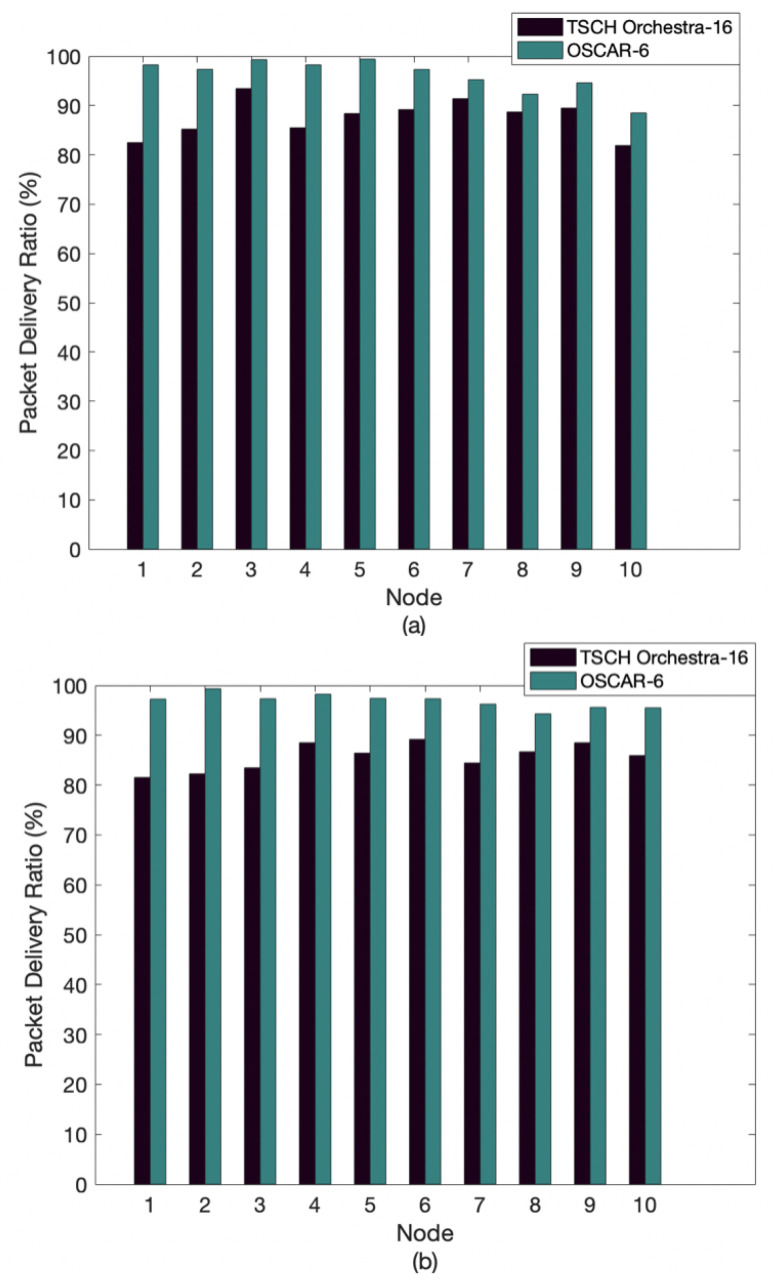
Average packet delivery ratio of OSCAR and Orchestra: (**a**) first topology and (**b**) second topology.

Figure 7 illustrates the average packet delivery rate for OSCAR and Orchestra. In this experiment, each node sent 2000 packets to the root, for a total of 20,000 packets sent across the network. Additionally, the data rate has been set to 5 pkts/min. For the first and second topology, OSCAR has a better delivery rate compared to Orchestra. With OSCAR, we notice that nodes 8–10 have higher packet delivery rates when the topology is condensed. Indeed, these nodes are in class 3, whereas when the topology is more dispersed, these nodes belong to classes 4 and 5. For example, node 10 displays a rate of 88.5% for the first topology and 95.7% for the second topology. This demonstrates that the allocation of slots according to the class of the node plays an important role in the performance of the network. The packet delivery rate for Orchestra remains roughly equivalent in each of the two network layouts. This can be explained because Orchestra allocates the same number of slots to all nodes, regardless of the position of that node.

#### 5.2.2. Impact of Traffic Load

**Figure 8:**
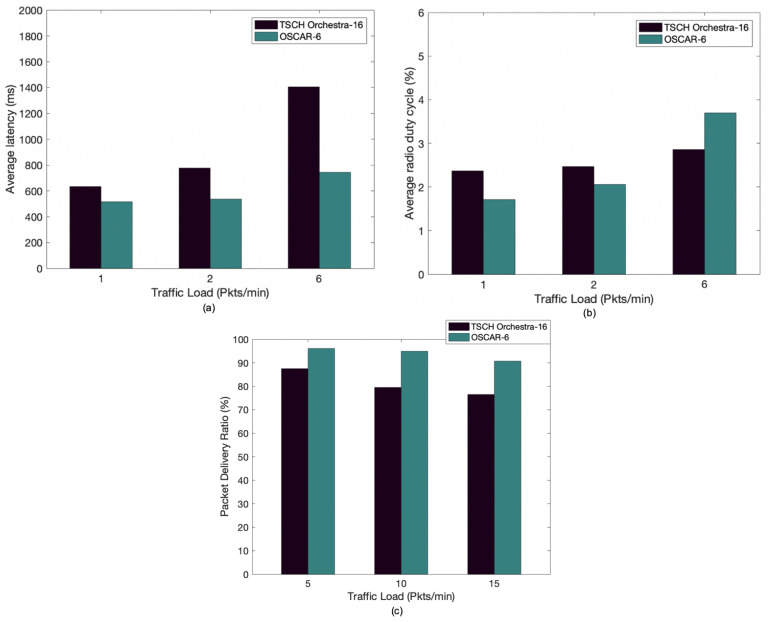
Comparison between Orchestra and OSCAR according to traffic load: (**a**) average end-to-end delay, (**b**) average radio duty cycle, and (**c**) average packet delivery ratio.

The purpose of this scenario is to test the behavior of the network when the nodes have more inbound traffic, more child nodes, or an overlapping schedule. We use the network layout of Figure 1. Three configurations for the data rate are used for latency and duty cycle, namely, 1, 2, and 6 pkts/min. Figure 8a shows that the latency performance is degraded for Orchestra as the traffic load increases. This is mainly due to the lack of transmission opportunities of nodes closer to the root. OSCAR reacts better to dense traffic and thus exhibits better latency overall by allocating more time slots as necessary. This is particularly notable in the 6 pkts/min scenario where the average latency is halved by using OSCAR.

The previous performance gains are achieved, as can be seen in Figure 8b, by slightly sacrificing power consumption in the highest traffic load scenario, as OSCAR grants more cells to the congested nodes. However, this increased consumption occurs only when the network is overloaded. We notice that OSCAR does not significantly increase the average duty cycle compared to the improvements achieved in latency. Finally, the average rate of packet delivery between OSCAR and Orchestra as a function of the data rate is plotted in Figure 8c. In this experiment, each node sent 2000 packets to the root, for a total of 20,000 packets sent across the network. In addition, a packet transmission intervals of 12 s, 6 s, and 5 s are used. It can be seen that Orchestra’s packet delivery performance is degraded as the traffic load increases. With a data rate of 5 pkts/min, OSCAR shows a result of 96.1% against 87.5% for Orchestra. When the packet transmission interval is increased to 15 pkts/min, Orchestra’s packet delivery ratio worsens considerably and is reduced to 76.4% compared to 90.7% for OSCAR. Better reliability is exhibited by OSCAR mainly due to the fact that it allots more unicast slots as the packets approach the sink.

### 5.3. Simulation Study

#### 5.3.1. Setup

The experimental study has limited number of nodes and in order to proceed with a larger-scale network performance characterization, simulations are used.

To show the performance of OSCAR relative to Orchestra in response to the traffic load, we study the performance by varying the number of nodes in the network within the simulation. To achieve this, we use COOJA, the network simulator distributed as part of the Contiki OS. The goal here is to see how OSCAR reacts to a large-scale network. To this end, we have considered a network where the nodes are deployed in a uniform grid topology. We have varied the number of nodes from 40 to 100 with a data rate equal to 1 pkts/min, and, as in the experiment, each node sent 2000 packets to the root.

#### 5.3.2. Impact of the Number of Nodes

**Figure 9:**
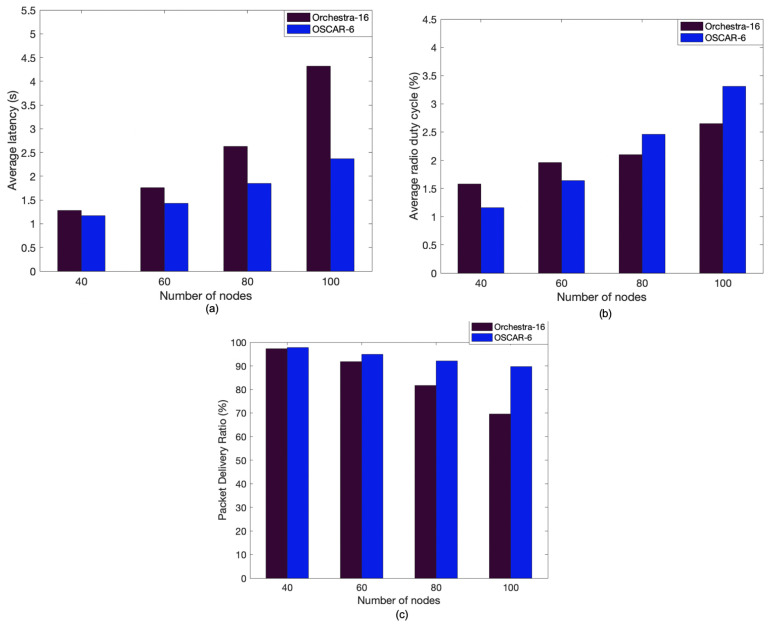
Comparison between Orchestra and OSCAR according to the number of nodes in the simulation: (**a**) average end-to-end delay, (**b**) average radio duty cycle, and (**c**) average packet delivery ratio.

Figure 9a compares the performance on the average latency between OSCAR and Orchestra by varying the number of nodes from 40 to 100. From this figure, we can clearly see that OSCAR outperforms Orchestra on average network latency. Thus, the greater the number of nodes in the network, the more the difference is apparent, corroborating the prior experimental results. OSCAR improves communication delay, and this observation can be justified by the fact that the most congested nodes are assigned more slots to evacuate the traffic. Indeed, the particularity of OSCAR is that the system grants more transmission opportunities to the nodes which need them most, namely, the nodes close to the root in the case of convergecast traffic.

Figure 9b shows the average duty cycle performance (and thus energy consumption) between OSCAR and Orchestra. We see that between 40 and 60 nodes the average duty cycle of the network is in favor of OSCAR. This can be explained because some nodes are less active than others and benefit from the reduction mechanism of slots allocated to inactive nodes introduced by OSCAR. On the other hand, the good performance of OSCAR, in terms of latency reduction, results in higher power consumption when the number of nodes reaches 80 and the traffic load becomes heavier. Finally, Figure 9c shows the average packet delivery ratio of Orchestra and OSCAR. As with the previous simulations, 2000 packets were sent by each node to the root. For a network of 60 nodes or less, the two systems have similar packet delivery performance, which shows that OSCAR maintains Orchestra’s good performance in terms of packet delivery. However, when the number of nodes is greater than 60, the gap becomes more pronounced and better results are observed for OSCAR compared to Orchestra. This observation can be explained by the fact that as the number of nodes increases, the traffic load also increases, causing packet loss at congested nodes. However, as seen previously, OSCAR minimizes this congestion by allocating more slots to these nodes, which results in a better delivery ratio.

Fundamentally, OSCAR shows that in larger networks, it can also bring significant latency and packet delivery advantages that widen as the network size is increased. OSCAR exhibits higher energy consumption in networks with significant amount of convergecast traffic (e.g., 80 nodes). However, the more drastic improvements in latency and packet delivery enabled by OSCAR as the number of nodes is increased offset the increased energy consumption.

## 6. Discussion

The experimental and simulation results have shown that OSCAR exhibits better performance with regards to end-to-end delay and packet delivery ratio compared to Orchestra. This is due to the better distribution of cells allocated to nodes introduced by Algorithm 1. Moreover, even with these gains, OSCAR does not significantly increase the average radio duty cycle. In fact, with Algorithm 2, the nodes save energy by switching off the inactive nodes. When the nodes are often active and transmitting data, the delay can be reduced because the additional slots that are allocated allow traffic to be evacuated. Whereas if the nodes become inactive, OSCAR starts to allocate fewer slots gradually and thus prevents the nodes from waking up more often. This limits the power consumption due to idle listening of nodes. Note that the gains that OSCAR exhibits are more significant when the traffic is dense and congested. However, for large-scale networks, there is a greater energy consumption for networks composed of 80 or more nodes.

WSNs have conflicting requirements to operate with both low power consumption and low latency. As networks are designed with low power considerations, the latency of the entire system typically increases. Conversely, for networks where the optimization priority is at the level of latency, greater energy consumption is generally observed. However, the advantage of OSCAR here is that the gains in latency and reliability are far more important than the minimal losses in energy suffered by the network. Moreover, energy gains can be seen in less active nodes, balancing the increased energy usage of the more congested nodes.

As seen previously, other solutions based on autonomous scheduling have been proposed. e-TSCH-Orch is one of these solutions and uses a strategy that when the slotframe has enough free time slots, the node can exploit them to empty its queue. However, this method aggravates the contention and collision problems because the nodes compete for free cells. For Escalator, nodes suffer more from idle listening overload, because when a node receives a packet, it immediately sends the received packet to its parent in the cell that follows consecutively. Idle listening is listed on all subtree nodes and amplifies wasted energy. ALICE allocates the same number of Tx/Rx slots in a slotframe under a directional link. However, as the traffic increases, ALICE also suffers from collisions leading to link losses. Compared to other solutions, OSCAR provides a concrete solution to the congestion problem present in data collection applications mainly around the sink. The main feature is that it distributes slots better for networks with convergecast traffic that suffer from severe latency problems when approaching the sink with other algorithms such as Orchestra. This is done with the distribution of nodes under different classes, introduced by OSCAR, which makes it possible to increase or reduce the number of slots allocated when the node is, respectively, near and far from the sink. With high traffic load, OSCAR exhibits a very low latency, at the cost of an additional energy consumption that is nevertheless mitigated by the mechanism of reduction of slots allocated to inactive nodes.

The OSCAR experimental results presented here show that the energy consumption of the nodes around the root is increased. Indeed, OSCAR arbitrarily allocates slots to nodes based on their position relative to the root, and a fully dynamic allocation based on the real-time network traffic signature would be worthwhile to explore. OSCAR does not provide a solution to the potential problem of cell mismatch when the transmitter has not yet become aware of a change. In this case, a retransmission happens on a different cell. A future improvement would be to introduce a mechanism to allow the management of the cell mismatch when the system has not yet become aware of the traffic change. It will be interesting in future work to join a combination of this work with artificial intelligence algorithms, for example, where nodes could adapt in real-time to the traffic load and signature by deciding to allocate or remove slots dynamically to nodes according to their immediate need, especially as the current global problem evolves with the capture of huge amounts of data.

## 7. Conclusions

In this paper, a new autonomous scheduling design, based on Orchestra, is proposed for convergecast TSCH networks. This algorithm, named OSCAR, manages the number of timeslots allocated to each node using the value of the rank described by the RPL routing protocol. The nodes are distributed under different layers where each node is assigned a classID based on the proximity to the root. The goal being that the closer the node is to the root, the more slots it gets in order to maximize the transmission opportunities. To avoid wasted energy, OSCAR sets up a mechanism to adjust the radio duty cycle of each node by reducing the slots allocated to inactive nodes regardless of their classID.

Performance analysis shows that OSCAR outperforms Orchestra on the average latency, especially when the traffic is heavy and congested. The performance of the Orchestra and OSCAR schedules was investigated by configuring various traffic intensities, ranging from 1 pkts/min to 6 pkts/min. The experimental results show that OSCAR reacts better to dense traffic and thus exhibits better latency overall by allocating more time slots when necessary, while Orchestra does not. Moreover, through simulations, the variation in the number of nodes has shown that OSCAR reacts better in large scale networks. As the network size increases and causes larger traffic load, OSCAR’s latency and packet delivery advantages widen in comparison to that of Orchestra. This widening of the performance advantages enabled by OSCAR comes at the cost of added energy consumption for networks composed of 80 or more nodes. Regarding the energy consumed, we have seen that the average OSCAR duty cycle depends, as does the latency, on the position of the node in the network. In the two arrangements tested in the experiments, this is lower than Orchestra when the traffic load is low, due to the service cycle adjustment algorithm. However, it becomes important as the network traffic load increases because the network is constantly in demand and the nodes are more active compared to Orchestra. Finally, the packet delivery rate remains similar between OSCAR and Orchestra, whatever the position of the node with low traffic. However, OSCAR shows better performance as the traffic load increases.

Fundamentally, this work paves the way for dynamic scheduling that attempts to reduce latency and packet delivery in congested convergecast scenarios, while reducing the impact on the network’s energy consumption in smaller networks. Overall, this work gives perspective on efficient cell distribution mechanisms and introduces a solution that can serve as a new scheduling method for convergecast networks.
